# The relationship of nerve fibre pathology to sensory function in entrapment neuropathy

**DOI:** 10.1093/brain/awu288

**Published:** 2014-10-17

**Authors:** Annina B. Schmid, Jeremy D. P. Bland, Manzoor A. Bhat, David L. H. Bennett

**Affiliations:** 1 Nuffield Department of Clinical Neurosciences, University of Oxford, OX3 9DU, Headington, UK; 2 School of Health and Rehabilitation Sciences, The University of Queensland, St Lucia, QLD 4072, Australia; 3 Department of Clinical Neurophysiology, Kent and Canterbury Hospital, CT1 3NG, Canterbury Kent, UK; 4 Department of Physiology, Centre for Biomedical Neuroscience, School of Medicine, University of Texas Health Science Centre, San Antonio, TX, USA

**Keywords:** entrapment neuropathy, carpal tunnel syndrome, skin biopsy, nodes of Ranvier, small fibres, quantitative sensory testing

## Abstract

Surprisingly little is known about the impact of entrapment neuropathy on target innervation and the relationship of nerve fibre pathology to sensory symptoms and signs. Carpal tunnel syndrome is the most common entrapment neuropathy; the aim of this study was to investigate its effect on the morphology of small unmyelinated as well as myelinated sensory axons and relate such changes to somatosensory function and clinical symptoms. Thirty patients with a clinical and electrophysiological diagnosis of carpal tunnel syndrome [17 females, mean age (standard deviation) 56.4 (15.3)] and 26 age and gender matched healthy volunteers [18 females, mean age (standard deviation) 51.0 (17.3)] participated in the study. Small and large fibre function was examined with quantitative sensory testing in the median nerve territory of the hand. Vibration and mechanical detection thresholds were significantly elevated in patients with carpal tunnel syndrome (*P < *0.007) confirming large fibre dysfunction and patients also presented with increased thermal detection thresholds (*P < *0.0001) indicative of C and Aδ-fibre dysfunction. Mechanical and thermal pain thresholds were comparable between groups (*P > *0.13). A skin biopsy was taken from a median nerve innervated area of the proximal phalanx of the index finger. Immunohistochemical staining for protein gene product 9.5 and myelin basic protein was used to evaluate morphological features of unmyelinated and myelinated axons. Evaluation of intraepidermal nerve fibre density showed a striking loss in patients (*P < *0.0001) confirming a significant compromise of small fibres. The extent of Meissner corpuscles and dermal nerve bundles were comparable between groups (*P > *0.07). However, patients displayed a significant increase in the percentage of elongated nodes (*P < *0.0001), with altered architecture of voltage-gated sodium channel distribution. Whereas neither neurophysiology nor quantitative sensory testing correlated with patients’ symptoms or function deficits, the presence of elongated nodes was inversely correlated with a number of functional and symptom related scores (*P < *0.023). Our findings suggest that carpal tunnel syndrome does not exclusively affect large fibres but is associated with loss of function in modalities mediated by both unmyelinated and myelinated sensory axons. We also document for the first time that entrapment neuropathies lead to a clear reduction in intraepidermal nerve fibre density, which was independent of electrodiagnostic test severity. The presence of elongated nodes in the target tissue further suggests that entrapment neuropathies affect nodal structure/myelin well beyond the focal compression site. Interestingly, nodal lengthening may be an adaptive phenomenon as it inversely correlates with symptom severity.

## Abstract (precis)

The impact of peripheral entrapment neuropathies on target innervation remains unknown. Using quantitative sensory testing, neurophysiology and skin biopsies, Schmid *et al.* demonstrate that carpal tunnel syndrome affects large fibres and their nodal complexes, but is also associated with a reduction in the number and functioning of small sensory axons.

## Introduction

Entrapment neuropathies are the most common peripheral neuropathies (Shiri, 2014). Their underlying pathophysiology is ascribed to an increased pressure on peripheral nerves (e.g. in the carpal tunnel; Gelberman *et al.*, 1981; Luchetti *et al.*, 1990), which leads to impaired neural microcirculation (Rydevik *et al.*, 1981) followed by focal demyelination (Mackinnon, 2002). In humans, diagnosis for entrapment neuropathies therefore largely relies on tests of myelinated fibre function, with neurophysiology playing a prominent role. Whereas the use of neurophysiology is recommended for decision-making in the management of some entrapment neuropathies (Alrawi *et al.*, 2007; AAOS, 2008), it correlates poorly with patients’ symptoms and level of disability (Mondelli *et al.*, 2000; Longstaff *et al.*, 2001).

We have recently shown in an animal model that mild nerve compression predominantly causes degeneration of small diameter axons; large diameter myelinated axons demonstrate demyelination at the site of compression but the axons remain structurally intact (Schmid *et al.*, 2013). There is preliminary evidence from clinical studies that the function of small nerve fibres may be affected in entrapment neuropathies. For instance, laser-evoked brain potentials are reduced (Arendt-Nielsen *et al.*, 1991) and sympathetic skin reflexes and vasomotor function decreased in the median nerve territory of patients with carpal tunnel syndrome (Wilder-Smith *et al.*, 2003; Kiylioglu, 2007). Results from quantitative sensory testing are more controversial with some authors reporting loss of function mediated by small myelinated or unmyelinated fibres in cervical radiculopathy or carpal tunnel syndrome (Chien *et al.*, 2008; Tamburin *et al.*, 2010; Tampin *et al.*, 2012) whereas others could not confirm thermal hypoaesthesia indicative of small fibre dysfunction (de la Llave-Rincon *et al.*, 2009).

Currently, the gold standard to diagnose small fibre involvement in neuropathies is the evaluation of target tissue of the affected nerves with skin biopsies (Lauria *et al.*, 2005). Specifically, the density of intraepidermal nerve fibres is a measure of the integrity of small diameter axons and has been shown to be reduced in different neuropathic pain conditions (Devigili *et al.*, 2008; Martinez *et al.*, 2010; Uceyler *et al.*, 2011). Skin biopsies can also be used to determine the integrity of large fibres and their myelin sheath (Provitera *et al.*, 2007; Myers *et al.*, 2013), a technique that has been successfully used in patients with demyelinating neuropathies (Doppler *et al.*, 2012, 2013).

Despite the high prevalence, surprisingly little is known about the impact of entrapment neuropathies on target innervation. To our knowledge, there is only one study evaluating the effect of entrapment neuropathy on cutaneous innervation. This investigated a sample of patients with carpal tunnel syndrome (Ramieri *et al.*, 1995), was purely descriptive with inconclusive results, and performed in relatively thin skin sections (15–30 μm), which does not allow adequate quantification of epidermal nerve fibres (Lauria *et al.*, 2010). Direct inferences on potential changes to target innervation in patients with entrapment neuropathies are therefore currently not possible.

The aim of this study was to investigate the effect of entrapment neuropathy on target innervation including evaluation of somatosensory function, neurophysiology and detailed morphological assessment of myelinated and unmyelinated sensory afferents. Furthermore, potential associations of these measures with patients’ symptoms and function were determined. To achieve this, carpal tunnel syndrome was used as a model because it is the most prevalent entrapment neuropathy and provides excellent access to both target tissue as well as the compression site for electrodiagnostic testing.

## Materials and methods

### Participants

Thirty patients who met electrodiagnostic (AANEM, 2002) and clinical (AAN, 1993) criteria for carpal tunnel syndrome were recruited through the local print media, advertisements on public notice boards and the neurophysiology clinic at the local hospital. Patients were excluded if electrodiagnostic findings were indicative of peripheral neuropathies other than carpal tunnel syndrome, if another medical condition that affected the upper extremity or neck was present, if a history of previous surgery or significant trauma to the upper limb or neck existed, or if carpal tunnel syndrome was related to pregnancy or diabetes. We identified an additional four patients who met the clinical criteria for carpal tunnel syndrome but had negative electrodiagnostic tests. These patients were not included in the cohort analysis but the results of their skin biopsy are reported separately. An age and gender matched cohort of 26 healthy volunteers without any systemic medical condition or a history of hand, arm or neck symptoms was included. The study was approved by the institutional ethics committee (reference number 10/H0706/35) and all participants gave informed written consent before participating.

Participants attended a single appointment during which one trained examiner performed electrodiagnostic tests, quantitative sensory testing and a skin biopsy.

### Symptom and function questionnaires

To determine the severity of symptoms and function impairment, patients with carpal tunnel syndrome completed the Boston Carpal Tunnel Questionnaire, which contains symptom and function components that are scored from 0 (no symptoms/disability) to 5 (very severe symptoms/disability) (Levine *et al.*, 1993). They also completed visual analogue scales (VAS, 0–10) for the level of pain, paraesthesia and numbness over the past 24 h and the Brief Pain Inventory, which contains a symptom severity subscore as well as a subscore on the interference of symptoms with the patient’s life (scores ranging from 0–10 with 10 indicating severe symptoms/interference) (Cleeland and Ryan, 1994). The Neuropathic Pain Symptom Inventory (Bouhassira *et al.*, 2004) was used to discriminate and quantify the following dimensions of neuropathic pain: superficial spontaneous pain (question 1), deep spontaneous pain (questions 2 and 3), paroxysmal pain (questions 5 and 6), evoked pain (questions 8, 9 and 10) as well as paraesthesia and dysaesthesia (questions 11 and 12) (Freeman *et al.*, 2014). In the Neuropathic Pain Symptom Inventory, the rating of each subscore can range from 0–10 and the total score from 0–100 with higher scores representing higher symptom severity. Participants also completed the pain catastrophizing scale (Sullivan *et al.*, 1995), the Depression Anxiety Positive Outlook Scale (DAPOS) (Pincus *et al.*, 2004) and the Insomnia Severity Index (ISI, range 0–28 with higher scores suggesting more severe insomnia) (Bastien *et al.*, 2001).

### Electrodiagnostic tests

Electrodiagnostic testing was performed with an ADVANCE system (Neurometrix) using conventional reusable electrodes. Hand temperature was standardized to >31°C. Orthodromic sensory nerve action potential latencies, amplitudes and nerve conduction velocities were recorded over the digit to wrist segments for the median (index finger), ulnar (little finger) and superficial radial nerve (snuffbox). Motor studies were performed for the median nerve (abductor pollicis brevis stimulated from the wrist and antecubital fossa), ulnar nerve (adductor digiti minimi stimulated from the wrist and elbow) and radial nerve (extensor indicis proprius stimulated from the spiral groove). We also recorded orthodromic sensory nerve action potentials by stimulating the ring finger and recording at the wrist. The presence of a ‘double peak’ (increased latency of median sensory nerve action potential compared to ulnar sensory nerve action potential) was considered abnormal (Uncini *et al.*, 1989). The motor latency difference between the median nerve (second lumbrical) and ulnar nerve (palmar interossei) were also measured over a fixed distance of 8 cm with a delay of the median motor potential relative to the ulnar latency >0.4 ms deemed abnormal (Preston and Logigian, 1992). Electrodiagnostic test severity was graded according to the criteria by Bland (2000) (Supplementary Table 1). Patients were only included if they had at least very mild electrodiagnostic findings (except for the four patients with negative tests, which are reported separately) whereas control participants were required to have no subclinical signs of carpal tunnel syndrome upon electrodiagnostic testing. During data analysis, absent sensory and motor recordings were replaced with values of zero for amplitudes (*n = *7 and *n = *1, respectively) but excluded from analysis of latencies and nerve conduction velocities to prevent inflated results.

### Quantitative sensory testing

Somatosensory phenotypes were determined according to a previously published protocol of the German research network of neuropathic pain (Rolke *et al.*, 2006). Cold and warm detection thresholds as well as cold and heat pain thresholds and thermal sensory limen were established using a quantitative sensory testing (QST) Thermotest (Somedic). We also tested mechanical detection and pain thresholds as well as mechanical pain sensitivity, pressure pain thresholds, wind up ratio and vibration detection thresholds. Participants were familiarized with the testing procedure on the dorsum of the non-experimental hand before all parameters were measured over the dorsum of the studied hand (innervated by the radial nerve) followed by the palmar side of the index finger (innervated by the median nerve). Pressure pain thresholds were recorded over the thenar eminence (median nerve territory) and the extensor digitorum muscle in the forearm (radial nerve territory) and vibration detection thresholds were tested over the ulnar styloid process (radial nerve territory) and on the palmar side of the distal end of the second metacarpal (median nerve territory). In patients with bilateral carpal tunnel syndrome, their most affected hand was tested, whereas we measured the non-dominant hand in control participants. All QST data were log-transformed to achieve a normal distribution and z-scores were calculated [z-score = (value of the participant − mean value of controls)/standard deviation of controls; Rolke *et al.*, 2006]. Positive z-scores represent gain of function whereas negative z-scores denote loss of function.

### Skin biopsy

A 3-mm diameter skin punch biopsy was performed on the ventro-radial side of the proximal phalanx of the index finger, an area innervated by the median nerve. In three patients, we refrained from taking a biopsy as they were medicated with warfarin. Subcutaneous anaesthesia was achieved with lidocaine (1%, 1–1.2 ml) before the biopsy was taken under sterile conditions. The biopsy was fixed in fresh periodate-lysine-paraformaldehyde (2%) for 30 min. Tissue was then washed in 0.1 M phosphate buffer and stored for 2–3 days in 15% sucrose in 0.1 M phosphate buffer. After embedding in O.C.T., the tissue was snap frozen and stored at −80°C. Fifty-micrometre thin sections were cut on a cryostat and double stained with myelin basic protein (MBP, Abcam, 1:500) and protein gene product 9.5 (PGP, Ultraclone, 1:1000) using a previously described free-floating method (Doppler *et al.*, 2013). In brief, sections were blocked using 5% fish gelatine before incubating overnight at 4°C with the primary antibodies. The next day, sections were washed in PBS containing 0.1% Triton™ X-100 and secondary antibodies were added overnight at 4°C (Cy3 Stratech, 1: 1000; Alexa Fluor® 488 Abcam, 1: 500). On the third day, sections were washed in PBS containing 0.1% Triton™ X-100 followed by PBS alone before they were mounted on slides for confocal analysis.

Intraepidermal nerve fibre density (IENFD) was determined by the same blinded observer on three sections per participant using an Axio LSM 700 microscope with an Observer Z1 imaging system (Zeiss). IENFD was quantified on PGP9.5 stained sections according to the current guidelines (Lauria *et al.*, 2010) and expressed as fibres per mm epidermis. Dermal innervation was evaluated by counting nerve bundles containing at least five PGP9.5^+^ axons per mm^2^ dermis (excluding nerves of the subepidermal plexus) as previously described (Doppler *et al.*, 2012). Dermal myelinated fibres were expressed as a percentage of PGP9.5^+^ nerve bundles containing MBP^+^ fibres (Uceyler *et al.*, 2013). We also quantified the number of Meissner corpuscles per mm epidermis. These mechanoreceptors can be found in glabrous skin and respond to low frequency stimuli including light touch and vibration (Provitera *et al.*, 2007). Nodes of Ranvier (≥15 per patient) and internodes were identified in the subepidermal plexus of MBP/PGP9.5 double-stained sections and their length determined with the Zen black software (Zeiss) on confocal image stacks taken at 2 μm intervals at ×40 magnification. In some participants, analysis of three sections was insufficient to reach the minimum threshold of 15 nodes. In this situation, up to a further four sections were stained and analysed again in a blinded manner. Based on a previous study using biopsies at the same location, nodes > 6.1 µm length were considered elongated (Doppler *et al.*, 2013). The fibre (MBP staining) and axon diameters (PGP 9.5 staining) of internodes in the subepidermal plexus that were visible in their entirety were determined at four randomly selected sites on the same images using ImageJ (NIH) (Nolano *et al.*, 2003). The G-ratio for each participant was expressed as the average of the mean axon/fibre ratios for each internode.

To determine potential changes in nodal architecture, the distribution of contactin-associated protein (Caspr; Bhat *et al.*, 2001; 1:1500), which is located at the paranodes (Rios *et al.*, 2000) and anti-pan-sodium channels (pNav, Sigma, 1:100), which detects a small band of voltage gated sodium channels (VGSC) in the node of Ranvier, was evaluated on sections triple stained with MBP (Chemicon, 1:500) using the same free-floating protocol as described above (secondary antibodies: Alexa Fluor® 488 and 568 Abcam, 1:1000; Alexa Fluor® 405 Abcam, 1:100). For each node of Ranvier in the subepidermal plexus of all patients and 13 control participants, we determined the length as well as the gap between Caspr staining and the extent of pNav staining on confocal image stacks taken at 2 -μm intervals at ×40 magnification. In the same images, we also determined the length of internodes present in the subepidermal plexus.

### Statistical analysis

SPSS Statistics Version 21 (IBM) was used for statistical analysis. All data were tested for normality with the Kolmogorov-Smirnov test and by visual inspection of their distribution. Mean values and standard deviations (SD) are reported for normally distributed data and median with interquartile range (IQR) for non-normally distributed data. QST z-scores and histological findings of patients with carpal tunnel syndrome and control participants were compared with independent samples *t*-tests and Mann-Whitney U-tests as appropriate. Spearman correlation analyses were performed to explore associations between histological parameters, QST findings, neurophysiological data as well as symptom and functional outcomes in patients with carpal tunnel syndrome. We used receiver operating curves (ROC) and area under the curve (AUC) to determine the diagnostic value of cut-offs for IENFD compared to electrodiagnostic testing. An AUC of <0.5 was classified as no discrimination, 0.7–0.8 as acceptable, 0.8–0.9 as excellent and an AUC ≥0.9 as outstanding discrimination (Hosmer and Lemeshow, 2000). Sensitivity (fraction of positive cases correctly classified) and specificity (fraction of negative cases correctly identified) as well as positive [sensitivity/(1 − specificity)] and negative likelihood ratios [(1 − sensitivity)/specificity] and their associated 95% confidence intervals (CI) were computed for IENFD. Significance was set at *P = *0.05.

## Results

The demographic and clinical baseline data are presented in Table 1. The mean age of patients with carpal tunnel syndrome was 56 years with a higher proportion of females (17 of 30 patients), which reflects the frequent onset of symptoms in middle age and higher prevalence in females (Nora *et al.*, 2004). The duration of carpal tunnel syndrome varied significantly ranging from 3 months to 20 years with a mean duration of 4.5 years. The median ratings of the symptom and function scales of the Boston carpal tunnel questionnaire (2.5 and 1.8, respectively), the Brief Pain Inventory (severity 2.75, interference 1.29) and the Neuropathic Pain Symptom Inventory (8.68) reflects a mild to moderate pain severity and functional impairment in our patients with carpal tunnel syndrome. This is comparable to a previous cohort (Schmid *et al.*, 2012). Patients were comparable to the control group in their ratings of the Pain Catastrophizing Scale and DAPOS (*P > *0.196), but had significantly higher ratings on the Insomnia Severity Index, which is in accordance with sleep impairment as a cardinal sign of carpal tunnel syndrome [median (IQR) patients with carpal tunnel syndrome: 9.00 (7.50); controls: 3.00 (6.25), *P = *0.001].

**Table 1:** Demographic and clinical data of patients with carpal tunnel syndrome and healthy volunteers

	Carpal tunnel syndrome	Controls	*P*-value
Gender (female/male)	17/13	18/8	0.073
Mean age, years (SD)	56.4 (15.3)	51.0 (17.3)	0.233
Mean weight, kg (SD)	79.7 (18.0)	73.3 (26.0)	0.053
Mean height, cm (SD)	166.6 (12.6)	161.9 (33.9)	0.342
Questionnaires			
Mean duration of symptoms, months (SD)	53.9 (55.0)		
Median VAS (IQR)			
Pain	2.0 (4.4)		
Numbness	2.5 (5.9)		
Paraesthesia	4.0 (6.1)		
Median Boston scale (IQR)			
Symptoms	2.5 (1.2)		
Function	1.8 (1.0)		
Median NPSI (IQR)	8.68 (14.75)		
Median Brief Pain Inventory (IQR)			
Severity	2.75 (3.00)		
Interference	1.29 (3.43)		

NPSI = Neuropathic Pain Symptom Inventory; VAS = Visual Analogue Scale.

Only a few patients with carpal tunnel syndrome took medications to relieve their hand symptoms. These included paracetamol (*n = *2), ibuprofen (*n = *1), piroxicam gel (*n = *1), and one patient took a combination of amitriptyline and codeine. Some patients were prescribed pain medication for other conditions (e.g. knee or hip osteoarthritis, depression) such as antidepressants (*n = *2), opiates (*n = *4), paracetamol (*n = *7) and non-steroidal anti-inflammatory drugs (NSAIDs, *n = *4). Of the control subjects, two subjects took NSAIDs and one subject took antidepressants.

Table 2 summarizes the electrodiagnostic test results in patients with carpal tunnel syndrome and control participants. According to the neurophysiological grading scale by Bland (2000), three patients had very mild, nine mild, ten moderate, two severe, five very severe and one patient had extremely severe carpal tunnel syndrome. The nerve conduction velocity of both the ulnar and radial nerves were within normal limits in all patients (>45 m/s in patients younger than 40 years old and >40 m/s in patients over 40 years old). Whereas the radial sensory nerve action potentials and CMAPs as well as ulnar sensory nerve action potentials were comparable between groups, the ulnar CMAPs tended to be slightly higher in patients with carpal tunnel syndrome.

**Table 2:** Neurophysiology data of patients with carpal tunnel syndrome and healthy volunteers

	Carpal tunnel syndrome	Controls	*P*-value
Median nerve			
SNAP (μV)	7.26 (5.88)	15.23 (14.82)	<0.0001
Sensory nerve conduction velocity (m/s)	35.10 (7.87)	51.60 (8.81)	<0.0001
Distal motor latency (ms)	4.59 (0.40)	3.13 (0.49)	<0.0001
CMAP (mV)	6.59 (5.15)	7.07 (3.13)	0.663
Ulnar nerve			
SNAP (μV)	7.36 (4.90)	9.75 (6.79)	0.064
Sensory nerve conduction velocity (m/s)	52.57 (6.80)	51.81 (8.38)	0.987
Distal motor latency (ms)	2.05 (0.00)	2.05 (0.00)	0.267
CMAP (mV)	7.72 (2.87)	6.93 (2.82)	0.043
Radial nerve			
SNAP (μV)	14.71 (11.00)	11.92 (13.00)	0.497
Sensory NCV (m/s)	51.20 (7.00)	47.28 (6.00)	0.093
Distal motor latency (ms)	1.46 (0.00)	1.60 (0.00)	0.418
CMAP (mV)	2.50 (1.00)	1.56 (1.00)	0.067

Data are presented as median (IQR).

SNAP = sensory nerve action potential; CMAP = compound motor action potential; NCV = nerve conduction velocity.

### Somatosensory phenotype in patients with carpal tunnel syndrome suggests a combination of small and large fibre sensory loss

**Figure 1:**
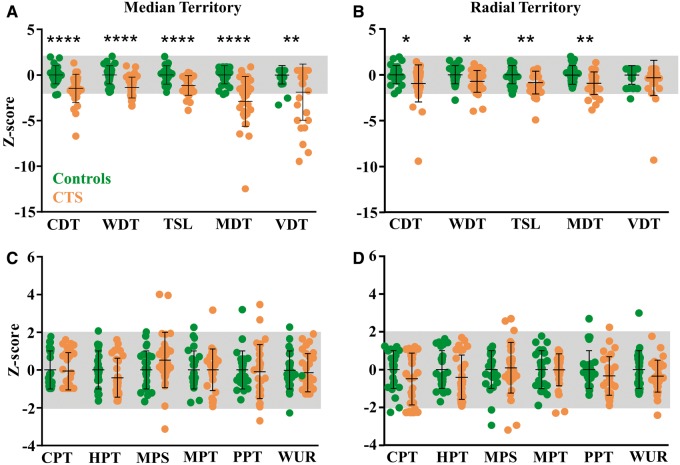
Somatosensory profiles determined with quantitative sensory testing in the median nerve territory (**A** and **C**) and the radial nerve territory (**B** and **D**) of patients with carpal tunnel syndrome (orange) and age and gender matched control subjects (green). Data are expressed as mean Z-scores with standard deviations and the grey area indicates the normal range of ± 2SD of the control subjects. (**A**) Patients with carpal tunnel syndrome have a significant loss of function in all detection thresholds measured in the median nerve territory compared to control subjects. (**B**) In the radial nerve territory, patients with carpal tunnel syndrome have loss of function in cold (CDT), warm (WDT) and mechanical detection thresholds (MDT) but not vibration detection (VDT). Patients also have a reduced ability to differentiate temperature changes (e.g. thermal sensory limen, TSL). No changes were apparent for any of the pain thresholds measured both in the median nerve territory (**C**) and the radial territory (**D**). This indicates that patients with carpal tunnel syndrome overall had a pattern of hypoaesthesia but not hyperalgesia. *P < 0.05, **P < 0.01, ***P < 0.001, ****P < 0.0001. CDT = cold detection threshold; WDT = warm detection threshold; TSL = thermal sensory limen; MDT = mechanical detection threshold; VDT = vibration detection threshold; CPT = cold pain threshold; HPT = heat pain threshold; MPS = mechanical pain sensitivity; MPT = mechanical pain threshold; PPT = pressure pain threshold; WUR = wind-up ratio; CTS = carpal tunnel syndrome.

Upon quantitative sensory testing in the median nerve territory, patients with carpal tunnel syndrome had significantly reduced cold and elevated warm detection thresholds (i.e. reduced sensitivity to warm and cool stimuli) as well as increased thermal sensory limens compared to control participants (all *P < *0.0001, Fig. 1A). This is indicative of loss of function mediated by C and Aδ fibres. As expected, patients with carpal tunnel syndrome also had significantly elevated mechanical detection and vibration detection thresholds (*P < *0.0001 and *P = *0.007 respectively) indicative of Aβ fibre involvement (Fig. 1A).

Mechanical (*P = *0.004), cold (*P = *0.036) and warm detection thresholds (*P = *0.022) as well as thermal sensory limen (*P = *0.007) were also altered in the radial nerve territory of the hand in patients with carpal tunnel syndrome albeit to a lesser extent than in the median nerve territory (Fig. 1B). Vibration detection thresholds in the radial nerve territory were comparable between groups (*P = *0.44).

No differences in mechanical and thermal pain thresholds were found between groups in both the median and radial nerve territory (*P > *0.13, Fig. 1C and D). There was also no difference in mechanical pain sensitivity or wind up ratio (*P > *0.129). No participant presented with a dynamic mechanical allodynia and only one control participant reported a paradoxical heat sensation. Overall, the somatosensory phenotype of patients with carpal tunnel syndrome reflects hypoaesthesia in both large and small fibre mediated domains.

### Patients with carpal tunnel syndrome have histological evidence of small fibre loss and lengthened nodes of Ranvier

#### Intraepidermal nerve fibre density

**Figure 2:**
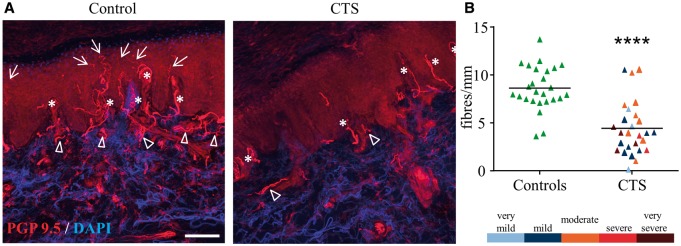
Patients with carpal tunnel syndrome have a reduced intraepidermal nerve fibre density. Images in (**A**) show representative skin biopsy sections from an age matched control participant (*left*) and a patient with carpal tunnel syndrome (CTS, *right*). Whereas there is an abundance of axons (red, protein gene product, PGP9.5) in the subepidermal plexus (arrow heads), the papillae (asterisks) and the epidermis (arrows) in the control skin, the skin of the patient with carpal tunnel syndrome shows a reduced density of axons in the subepidermal plexus and a clear reduction of axons piercing into the epidermis. Scale bar = 100 µm. The graph in **B** shows the mean intraepidermal nerve fibre density expressed as fibres per mm epidermis as well as single data points. Patients with carpal tunnel syndrome have a significantly lower intraepidermal nerve fibre density than the matched control group. This reduction was independent of electrodiagnostic test severity (colour coded according to the bar below). ****P < 0.0001.

PGP 9.5 staining revealed a dense subepidermal plexus in control participants with regular intrapapillary fibres. In contrast, the subepidermal plexus of patients with carpal tunnel syndrome appeared less dense but intrapapillary nerve fibres were present (Fig. 2A). There was a striking reduction in IENFD in the median nerve territory in patients with carpal tunnel syndrome [mean (SD) fibres per mm: 4.5 (2.9)] compared to controls [8.7 (2.4), *P < *0.0001, Fig. 2B], implying a loss of small nerve fibres. This loss was independent of electrodiagnostic test severity (*P = *0.941), but a trend towards a weak correlation with symptom duration was identified (r = 0.379, *P = *0.051). The analysis of the receiver operating characteristic curve demonstrated an excellent discrimination of IENFD between participants with and without carpal tunnel syndrome as determined with clinical and electrodiagnostic criteria [AUC = 0.879 (lower and upper confidence interval 0.78 and 0.98, respectively), *P < *0.0001, Supplementary Fig. 1]. A cut-off of ≤7.1 fibres per mm revealed a sensitivity of 88.9% and a specificity of 84.6% with a positive likelihood ratio of 5.77 and a negative likelihood ratio of 0.13. Applying this cut-off in the current study, 25 patients with electrodiagnostically confirmed carpal tunnel syndrome (83.3%) had a reduced intraepidermal nerve fibre density whereas only four control participants (15.4%) fell below this cut-off limit.

#### Dermal fibres

**Figure 3:**
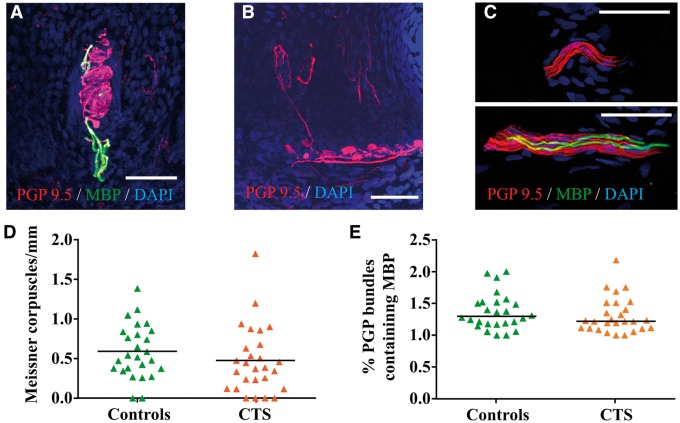
Representative images of dermal histology in 50 µm thick immuno-stained sections. (**A**) A Meissner corpuscle located in the dermal papilla. The characteristic winding-like structure of the Meissner corpuscle is formed by axons (red, protein gene product, PGP9.5) and it is innervated by a myelinated fibre (green, myelin basic protein, MBP). Nuclei are stained in blue (DAPI). (**B**) Example of a Merkel cell complex (red, PGP9.5) located at the base of the epidermis and innervated from fibres originating from the subepidermal plexus (bottom). (**C**) Representative images of the quantified dermal axon bundles containing more than five axons (red, PGP9.5) in the same section. Whereas the bundle on the top only contains unmyelinated fibres, on the bottom is an example of a dermal nerve bundle that contains two myelinated fibres (green, MBP). Calibration of 50 µm applies throughout. (**D**) Graph shows a comparable amount of Meissner corpuscles per mm epidermis in patients with carpal tunnel syndrome and controls participants. Data are shown as mean and single data points, *P = *0.28. (**E**) Graph shows comparable percentage of dermal PGP bundles containing MBP-positive fibres between patients with carpal tunnel syndrome and control participants. Plot depicts median and single data points, *P = *0.38.

For large nerve fibres, we found a comparable histology of Meissner corpuscles, most of which were innervated by a myelinated fibre (Fig. 3A). These corpuscles were regularly distributed in the papillaries along the epidermis. Upon quantification, we found no difference in the number of Meissner corpuscles per mm epidermis [mean (SD) carpal tunnel syndrome: 0.50 (0.44); controls: 0.58 (0.35), *P = *0.28, Fig. 3D]. We also found the occasional Merkel cells in both patients with carpal tunnel syndrome and control participants with comparable morphology (Fig. 3B). These slowly adapting mechanoreceptors were found at the base of the dermal papillae but as previously reported (Provitera *et al.*, 2007), their distribution was irregular and did not allow quantification.

In the dermis, the number of PGP^+^ bundles per mm^2^ were also comparable between groups [median (IQR) carpal tunnel syndrome: 3.91 (2.62); controls: 4.86 (2.93), *P = *0.07]. Brightly stained myelinated fibres were present in some of these dermal nerve bundles in both patients with carpal tunnel syndrome (Fig. 3C) and control participants with a comparable percentage of PGP^+^ dermal bundles containing MBP^+^ fibres [median (IQR) carpal tunnel syndrome: 1.22 (0.38); controls: 1.31 (0.35), *P = *0.38, Fig. 3E].

#### Myelin integrity and nodes of Ranvier

**Figure 4:**
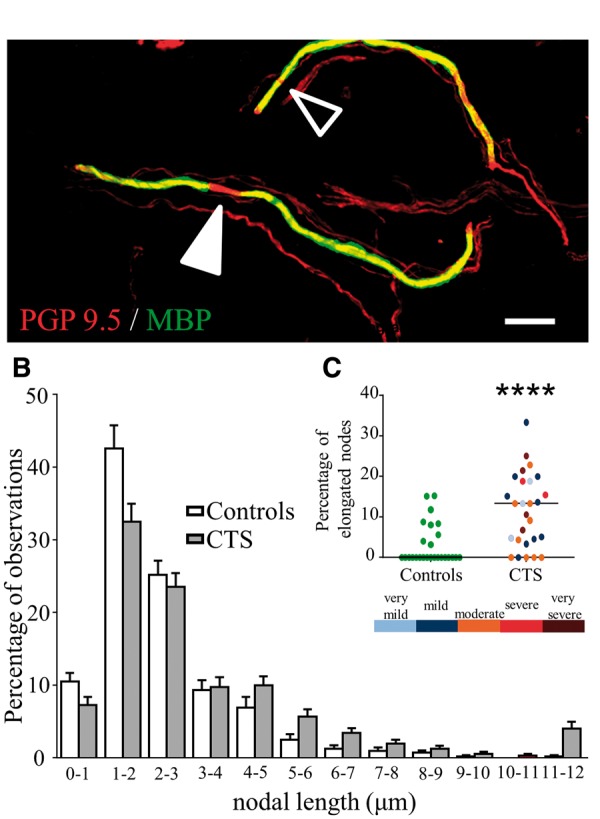
Patients with carpal tunnel syndrome have a higher proportion of elongated nodes. (**A**) A myelinated fibre (red, protein gene product, PGP9.5; green, myelin basic protein, MBP) with two nodes (arrow heads). The filled arrowhead points towards a clearly elongated node whereas the black arrowhead shows a normal nodal length. Scale bar = 20 µm. (**B**) The graph shows the percentage frequency histogram of nodal lengths in patients with carpal tunnel syndrome (grey) and control participants (white). There is a shift towards longer node lengths in patients with carpal tunnel syndrome (Kolmogorov-Smirnov *P < *0.0001). (**C**) The graph confirms that the proportion of elongated nodes >6.1 µm is higher in patients with carpal tunnel syndrome compared to control participants (*P < *0.0001) and is independent of electrodiagnostic test severity (colour-coded according to bar below).

We also looked more closely into the features of myelinated fibres firstly by evaluating the length of nodes of Ranvier in the subepidermal plexus [mean (SD) of 21.2 (7.9), range 15–53 nodes per participant]. We found a significant increase in nodal length in patients with carpal tunnel syndrome [median (IQR) carpal tunnel syndrome: 3.30 (1.30); controls; 2.37 (0.74), *P < *0.0001]. This difference was caused by a shift in the frequency distribution towards longer nodes in patients with carpal tunnel syndrome (Fig. 4A and B, Kolmogorov Smirnov *P < *0.0001). A greater number of patients displayed elongated nodes >6.1 μm length [median (IQR) carpal tunnel syndrome: 13.33 (14.40)] compared to controls [0.00 (6.17), *P < *0.0001, Fig. 4C]. The elongated nodes were found in different fibres within the biopsy and could also be present in a fibre with adjacent nodes of normal length. There was no correlation between nodal length or percentage of elongated nodes with disease stage (duration of symptoms: *P > *0.668; electrodiagnostic test severity: *P > *0.389). No significant differences were found between groups for internodal length [mean (SD) carpal tunnel syndrome: 82.49 (17.56); controls: 85.70 (19.79), *P = *0.629] or G-ratios [mean (SD) carpal tunnel syndrome: 0.78 (0.10); controls: 0.82 (0.10), *P = *0.166], which were within the range of previously reported values (Nolano *et al.*, 2003).

**Figure 5:**
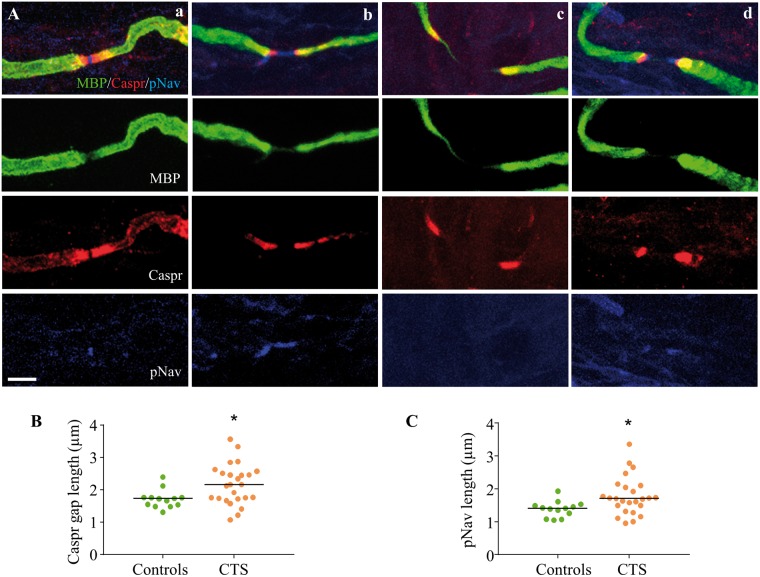
Elongated nodes are characterized by lengthening of the paranodal gap and dispersion of voltage gated sodium channels. (**A**) Merged images (first row) of nodes of Ranvier triple stained with MBP (green, second row), Caspr (red, third row) and pNav (blue, fourth row) of a control participant (a) and patients with carpal tunnel syndrome (b–d). Panel (a) depicts a normal node of a control participant with clear demarcation of Caspr staining in the paranodes as well as a short band of pNav staining; and (b) shows an elongated node with separation of caspr staining and dispersion of VGSCs within the nodal gap. This was the most frequently identified pattern. We also found occasional elongated nodes with a separation of Caspr staining and absent pNav staining (c). We also identified some heminodes (d), characterized by pNav staining adjacent to the paranodes with a pNav negative gap in the middle of the node. The calibration of 5 µm applies throughout. (**B**) shows the mean length of the Caspr gap in patients with carpal tunnel syndrome (orange) and control participants (green). This gap was significantly elongated in patients with carpal tunnel syndrome (*P = *0.021). (**C**) The mean length of nodal pNav staining was significantly higher in patients with carpal tunnel syndrome compared to control participants (*P = *0.026).

The architecture of nodes of Ranvier in control participants was characterized by clearly defined and regular Caspr staining at the paranodes with a small band of pNav staining located in the nodal gap [Fig. 5A(a)]. Caspr staining was also visible as fine lines along the axons representing the mesaxon. In patients with carpal tunnel syndrome, we observed nodes of both normal and elongated length. The nodes of normal length appeared to have a normal nodal architecture with clearly defined Caspr and pNav staining. In contrast, the elongated nodes were characterized by a clear increase in separation between the paranodes defined by the Caspr staining [mean µm (SD) gap between Caspr staining in controls: 1.71 (0.28); carpal tunnel syndrome: 2.16 (0.64), *P = *0.021, Fig. 5B], whereas the length of the paranodes seemed to be preserved [mean µm (SD) controls: 3.47 (0.39); carpal tunnel syndrome: 3.32 (0.56), *P = *0.395] and mostly symmetrical bilaterally [*P > *0.43, Fig. 5A(b–d)].

Within the elongated nodes, VGSC staining was dispersed between paranodes [Fig. 5A(b)] and appeared to be fainter and in some circumstances was even absent [(Fig. 5A(c)] compared to the distinct pNav^+^ band in normal nodes. We also observed occasional hemi-nodes, where a short band of pNav staining was located adjacent to the paranodes whereas the middle of the node did not contain VGSCs [Fig. 5A(d)]. Despite the presence of many nodes of normal length, statistical comparison confirmed a dispersion of pNav staining in patients with carpal tunnel syndrome [mean µm (SD): 1.78 (0.59)] compared to control participants [1.39 (0.24), *P = *0.026, Fig. 5C].

#### Patients with symptoms of carpal tunnel syndrome but normal neurophysiology

Interestingly, three of the four participants with symptoms highly suggestive of carpal tunnel syndrome but normal neurophysiology also had a reduced IENFD (fibres per mm in Patient A: 10.29; Patient B: 3.10; Patient C: 4.10; Patient D: 3.56), but similar numbers of Meissner corpuscles (Patient A: 0.17; Patient B: 1.23; Patient C: 1.11, Patient D: 0.77) and PGP^+^ dermal bundles (Patient A: 7.45; Patient B: 3.86; Patient C: 4.20; Patient D: 2.75) compared to the control participants (Supplementary Fig. 2). Similar to our carpal tunnel syndrome cohort, three patients with negative neurophysiology displayed an elevated percentage of elongated nodes >6.1μm (Patient A: 25.0%; Patient B: 2.4%; Patient C: 17.7%, Patient D: 0%). These preliminary findings indicate that histological changes to small axons and potentially nodes of Ranvier are apparent even if electrodiagnostic testing is negative and may reflect changes in the small cohort of patients in whom carpal tunnel syndrome is present but neurophysiology is negative.

### Correlation of histology, somatosensation and neurophysiology with patients’ symptoms and function

Table 3 details the correlation coefficients of histology, quantitative sensory testing and neurophysiology with patient symptom and function scales. There was no correlation between IENFD, Meissner corpuscles or the percentage of PGP bundles containing MBP with clinical symptoms. Surprisingly to us, the proportion of elongated nodes was inversely correlated with several pain related measures including the Brief Pain Inventory (pain interference subscale: r = −0.519, *P = *0.007 and pain severity subscale r = −0.482, *P = *0.013), the presence of paroxysmal pain as determined with the Neuropathic Pain Symptom Inventory (r = −0.521, *P = *0.006) as well as the Boston carpal tunnel questionnaire (symptom subscale r = −0.435, *P = *0.0023; function subscale r = −0.478, *P = *0.012). In contrast to our expectations, patients with more elongated nodes therefore seem to have less severe symptoms and reduced functional deficit and this was consistent across different validated scales.

**Table 3:** Spearman correlation coefficients (*P*-values) of histology, somatosensory phenotype and neurophysiology with patients symptoms and function

	Histology				Somatosensation	Neurophysiology median nerve	
	IENFD	Meissner	PGP/MBP	Elongated nodes	QST	SNAP	SNCV
Boston symptoms	NS	NS	NS	−0.435 (0.023)	NS	NS	NS
Boston function	NS	NS	NS	−0.478 (0.012)	NS	NS	NS
BPI pain	NS	NS	NS	−0.482 (0.013)	NS	NS	NS
BPI interference	NS	NS	NS	−0.519 (0.007)	NS	NS	NS
NPSI total	NS	NS	NS	NS	NS	NS	NS
NPSI paroxysmal	NS	NS	NS	−0.521 (0.006)	NS	NS	NS

NS = not significant; SNAP = sensory nerve action potential; SNCV = sensory nerve conduction velocity; QST = quantitative sensory testing; BPI = Brief Pain Inventory; NPSI = Neuropathic Pain Symptom Inventory.

Somatosensation as measured with QST did not correlate with patients’ symptoms or functional deficit (*P > *0.062). Similarly, both the motor and sensory neurophysiological parameters of the median nerve did not correlate with symptomatology (*P > *0.102). This is consistent with previous reports that have suggested little correlation between neurophysiological measures and clinical symptoms in patients with carpal tunnel syndrome (Mondelli *et al.*, 2000).

Within the histological findings, we identified a moderate correlation of intra-epidermal nerve fibre density with the density of Meissner corpuscles (r = 0.454, *P = *0.017) and PGP^+^ dermal nerve bundles (r = 0.405, *P = *0.036). There was however no correlation between intraepidermal nerve fibre density with any measure of myelination (percentage of myelinated dermal bundles *P = *0.471; internodal length *P = *0.201; percentage of elongated nodes *P = *0.298). No correlations were identified between the percentage of elongated nodes and any QST measure (all *P > *0.190).

## Discussion

The findings of this study confirm that focal nerve compression does not exclusively affect the myelinated, but also the unmyelinated nerve fibre population. This was not only apparent by loss of function of warm detection mediated by C-fibres, but also histologically by a striking loss of IENFD. We also provide evidence that nerve entrapment affects the nodal complex well beyond the lesion site as apparent by the presence of elongated nodes with altered VGSC distribution in axons distal to the compression site. The inverse relationship between the percentage of elongated nodes and patients’ symptoms and function deficit suggests that this may be an adaptive phenomenon.

It is commonly accepted that entrapment neuropathies predominantly affect the myelinated fibre population. This assumption is however based on evidence from animal models, which represent a more acute injury with substantial axon loss (Basbaum *et al.*, 1991; Hu *et al.*, 2007). Clinical tests consistently confirm a dysfunction of the large fibre population in entrapment neuropathies (Lundborg *et al.*, 1992; Padua *et al.*, 1997; Ginanneschi *et al.*, 2006; Tucker *et al.*, 2007). There is growing evidence from laser-evoked potentials (Arendt-Nielsen *et al.*, 1991), sympathetic testing (Wilder-Smith *et al.*, 2003; Kuwabara *et al.*, 2008) and QST (Chien *et al.*, 2008; Tamburin *et al.*, 2010) that the function of small fibres may also be affected. The comprehensive QST findings of this study confirm a dysfunction of both small and large fibres which is characterized by hyposensitivity to sensory stimuli (i.e. loss of function). Some authors have reported hypersensitivity to noxious stimuli in carpal tunnel syndrome (e.g. reduced thermal and pressure pain thresholds) (de la Llave-Rincon *et al.*, 2009; Fernandez-de-las-Penas *et al.*, 2009) which we did not observe here. This difference may relate to recruitment patterns. For instance our patients were mainly derived from primary care and the community rather than secondary care.

Unexpectedly, loss of function mediated by large and small nerve fibres was not only present in the median nerve territory, but also in the radial nerve area in the same hand albeit to a lesser extent. Although we cannot dismiss the possibility that carpal tunnel syndrome is an early manifestation of an otherwise subclinical systemic condition causing a polyneuropathy and hence impacting on sensory function of the radial nerve, this seems unlikely in our cohort: we carefully excluded patients with systemic conditions and neurophysiological parameters of the ulnar and radial nerves were normal. Ongoing paraesthesia was a major symptom in our cohort and may have acted as a distractor during threshold testing in the radial territory partially explaining these small differences. The somatosensory cortical representation of median, ulnar and radial innervated digital areas is less distinct in patients with carpal tunnel syndrome (Druschky *et al.*, 2000; Napadow *et al.*, 2006), a phenomenon which could also contribute to the greater difficulty in differentiating sensory stimuli in areas adjacent to the compressed nerve territory.

There have been very few studies of nerve fibre pathology as a consequence of human entrapment neuropathies (Foix and Marie, 1913; Thomas and Fullerton, 1963; Neary, 1975; Mackinnon *et al.*, 1986; Ramieri *et al.*, 1995). To summarize these previous studies, they are in broad agreement that nerve entrapment results in segmental demyelination followed by remyelination at the site of entrapment. There is much less agreement as to the presence and extent of distal axon loss although this is acknowledged for myelinated fibres in the most severe cases (Thomas and Fullerton, 1963; Mackinnon *et al.*, 1984*a*). The fate of unmyelinated axons remains largely unclear (Mackinnon *et al.*, 1986; Ramieri *et al.*, 1995).

To clarify the effect of entrapment neuropathy on distal sensory fibres and associated cells (e.g. Meissner corpuscles) and to relate this to symptoms, we performed a detailed analysis of cutaneous innervation within the median territory. Patients with carpal tunnel syndrome demonstrated a marked (50%) reduction of IENFD, which complemented the findings from QST in which we had detected changes in warm and cold detection thresholds. Conduction in unmyelinated nerve fibres is not assessed by conventional nerve conduction studies and indeed the reduction in IENFD was independent of the overall electrodiagnostic test severity although we noted a correlation between IENFD and the median sensory nerve action potential amplitude (r = 0.523, *P = *0.005). The comparable density of Meissner corpuscles and dermal PGP bundles suggest that the structural integrity of myelinated axons is largely preserved in entrapment neuropathies. This differentiates entrapment neuropathies from patients with other peripheral neuropathies where a reduction in Meissner corpuscles has been identified [e.g. diabetic neuropathy (Peltier *et al.*, 2013), Charcot–Marie–Tooth disease (Saporta *et al.*, 2009)]. Future studies are required to confirm these distinct histological changes in patients with other entrapment neuropathies such as cubital tunnel syndrome.

The aim of this study was to use skin biopsy to understand the pathophysiology of entrapment neuropathies rather than to develop a new diagnostic test. However, using combined clinical criteria and electrophysiology as a gold standard, a cut-off of ≤7.1 fibres per mm epidermis revealed a sensitivity of 88.9% and a specificity of 84.6%. Future studies are needed to determine the diagnostic accuracy and performance of skin biopsies in differentiating patients with actual carpal tunnel syndrome in a sample of patients in which the syndrome is suspected. Interestingly, three of four patients who had typical symptoms of carpal tunnel syndrome but normal neurophysiology demonstrated reduced IENFD. Previous studies report a small cohort of patients with clinically diagnosed carpal tunnel syndrome who have normal nerve conduction studies (Witt *et al.*, 2004; Koyuncuoglu *et al.*, 2005). Assessment of IENFD may complement other techniques such as ultrasound of the median nerve in investigating these patients (Koyuncuoglu *et al.*, 2005).

The here observed reduction in IENFD and the hyposensitivity on QST demonstrated here and by others (Tamburin *et al.*, 2010) unequivocally demonstrates small fibre dysfunction in carpal tunnel syndrome despite the experimental findings that unmyelinated axons are relatively resistant to acute compression (Dahlin *et al.*, 1989). Findings from a recent animal model mimicking chronic compression neuropathies also shows small fibre injury as determined by the evaluation of cell body areas and expression of ATF3 in dorsal root ganglia neurones (Schmid *et al.*, 2013). A working model of the pathophysiology of carpal tunnel syndrome has been that in mild/moderate cases symptoms are mainly secondary to increased pressure/ischaemia resulting in demyelination and conduction block at the compression site and only as the condition progresses to a more severe form is there axon degeneration. This has mainly been based on assessment of large fibre function using neurophysiology (Cappelen-Smith *et al.*, 2003; Sohn *et al.*, 2011). We would suggest, however, that distal degeneration of small fibres is a common consequence of carpal tunnel syndrome even in cases that would be labelled as ‘mild’ on nerve conduction studies. This could have prognostic implications as in some cases significant axon regeneration/collateral sprouting would be required to restore cutaneous innervation to normal levels following surgical release.

We did not observe a reduction in the number of nerve bundles in the dermis, the proportions of these bundles containing myelinated axons or in the number of Meissner corpuscles suggesting that there was no gross loss of large diameter myelinated axons. However, there was a clear change in the nodal complex as evident by a higher percentage of elongated nodes in patients with carpal tunnel syndrome. Elongated nodes have previously been identified in dermal myelinated fibres in patients with chronic inflammatory demyelinating polyradiculoneuropathy (Saporta *et al.*, 2009; Doppler *et al.*, 2013). Not all demyelinating neuropathies are associated with lengthened nodes of Ranvier: analysis of skin biopsies obtained from patients with Charcot–Marie–Tooth type 1A demonstrated nodes of normal length but shortened internodes (Saporta *et al.*, 2009). The distribution of the molecular components of the nodal complex in carpal tunnel syndrome was distinct to previously described patterns in human dermal myelinated fibres. In chronic inflammatory demyelinating polyradiculoneuropathies, nodes of Ranvier were elongated and paranodes identified by Caspr and neurofascin immunoreactivity were dispersed (Doppler *et al.*, 2013) whereas Caspr staining was fainter in patients with Guillain–Barré syndrome (Ruts *et al.*, 2012). In carpal tunnel syndrome, we noted preserved paranodal length with a widening of the nodal gap between the Caspr immunoreactive paranodes. Within this widened nodal gap, VGSCs were more diffusely distributed rather than being in a clearly demarcated band as was previously observed in chronic inflammatory demyelinating polyradiculopathies and Charcot–Marie–Tooth type 1A (Saporta *et al.*, 2009; Doppler *et al.*, 2013). It should be noted that the altered distribution of VGSCs was restricted to widened nodes of Ranvier and often in the same section and even the same fibre, nodes of Ranvier of normal width were seen with a normal distribution of Caspr and VGSCs.

In experimental models, both acute (Ochoa *et al.*, 1971; Dyck *et al.*, 1990) and chronic nerve compression (Mackinnon *et al.*, 1984*b*) interfere with the normal architecture of nodes of Ranvier and their adjacent paranodes at the compression site, presumably by ischaemia (Nukada *et al.*, 1985) and mechanical deformation of Schwann cells (Pham and Gupta, 2009; Lin *et al.*, 2012). Our findings demonstrate however that such changes are not confined to the entrapment site but significantly altered axoglial relationships are confirmed in distal innervation targets.

The mechanisms behind these nodal changes beyond the injury site remain speculative. We have recently identified demyelination over a substantial distance downstream of an experimental nerve compression site (Schmid *et al.*, 2013). Although there may be a secondary inflammatory cell infiltrate (Schmid *et al.*, 2013), entrapment neuropathies are not driven by an autoimmune/inflammatory process such as present in chronic inflammatory demyelinating polyradiculopathies. In experimental models of chemical demyelination and remyelination, disruption of the nodal complex has clearly been demonstrated (Arroyo *et al.*, 2004). However, we did not observe segmental demyelination (which has previously been reported in chronic inflammatory demyelinating polyradiculopathies; Saporta *et al.*, 2009). We do not have any firm evidence therefore that nodal lengthening is a consequence of segmental demyelination/remyelination. Another possible mechanism leading to node lengthening may be attributed to chronic mechanical forces acting on nervous tissue. It has previously been shown that chronic nerve stretching as induced during surgical limb lengthening leads to paranodal demyelination with subsequent node lengthening in animals (Abe *et al.*, 2004; Ichimura *et al.*, 2005). Interestingly, such mechanical node elongation induces a comparable pattern of VGSC dispersion (Ichimura *et al.*, 2005). Potentially, the previously reported relative immobility of the median nerve inside the carpal tunnel (Hough *et al.*, 2007; Korstanje *et al.*, 2012) may augment stretch forces acting on digital nerve branches during finger and wrist movements thus leading to paranodal demyelination.

We correlated several clinical and histological measures with questionnaires that are designed to tease out symptom clusters specifically in patients with carpal tunnel syndrome or neuropathic pain. Unexpectedly, the percentage of elongated nodes was the only parameter that correlated with patients’ symptoms across a number of different symptom/function scores. This has never before been demonstrated in any neuropathy and contrasts to IENFD, QST and nerve conduction studies, none of which correlated with symptoms or function. Interestingly, the inverse correlation between the frequency of elongated nodes and symptom severity implies a protective phenomenon. Conduction slowing or block due to increased neural tension (Wall *et al.*, 1992), elongated nodes (Ikeda *et al.*, 2000) and dispersion of VGSCs (Ichimura *et al.*, 2005) may explain a reduction in positive sensory phenomena such as paroxysmal pain and paraesthesia. Hypothetically, those patients who adaptively react with nodal elongation due to chronic nerve stretching may reduce the mechanical forces acting on their digital branches during hand and finger movements, which may explain a decrease in symptomatology followed by improved function.

To summarize, we have undertaken a detailed investigation of the impact of carpal tunnel syndrome on cutaneous innervation distal to the entrapment site, relating this to symptoms, somatosensory function and nerve conduction studies. We show for the first time that entrapment neuropathies have clear effects on both unmyelinated and myelinated fibres distal to the compression site. We show loss of small fibre function (upon QST) as well as marked structural compromise (reduced IENFD) that is independent of electrodiagnostic test severity. The presence of elongated nodes with VGSC dispersion >9 cm distal to the compression site further suggests that entrapment neuropathies affect the nodal complex in myelinated sensory fibres well beyond the focal lesion site. The presence of these elongated nodes is associated with a reduction in the severity of sensory symptoms and improved functional status, suggesting an adaptive mechanism.

## Abbreviations

IENFD: intraepidermal nerve fibre density
QST: quantitative sensory testing
VGSC: voltage gated sodium channel
